# Using a Pharmacy-Based Surveillance System to Improve Standards for TB Care in Kerala, India

**DOI:** 10.9745/GHSP-D-21-00346

**Published:** 2021-12-31

**Authors:** Rakesh PS, Shibu Balakrishnan, Rakesh Ramachandran, Smitha Nandhan, Nidhish Issac Samuel, Pramodkumar PP, Suja Aloysius

**Affiliations:** a World Health Organization National TB Elimination Program Technical Support Network, State TB Cell, Thiruvananthapuram, India.; b Project JEET, State TB Cell, Thiruvananthapuram, India.; c District TB Center, Kerala State Health Services, Kerala, India.

## Abstract

**Introduction:**

Eleven anti-TB drugs were included in the Government of India's Schedule H1 drug regulations in 2014. The National Strategic Plan for TB Elimination in India 2017–2025 recognized the opportunity to strengthen the TB surveillance system and improve the quality of TB care by implementing the Schedule H1 regulation. However, there were no documented systematic large-scale efforts to use Schedule H1 regulation to support TB surveillance or improve the quality of care. We aimed to document the process of implementation of the Schedule H1 regulation to enhance the quality of TB care and strengthen the TB surveillance system in Kerala, India.

**Methods:**

We conducted 33 in-depth interviews of the drugs control department enforcement officers, chemist shop owners, private-sector doctors, leaders of professional medical associations, and program managers and key staff of the TB Elimination Program in Kerala. Major themes identified were the process of implementation of Schedule H1 and how the National TB Elimination Program used the information. Findings from the qualitative interviews were corroborated with the quantitative information from the annual program performance reports and anti-TB drug sales data.

**Results:**

The TB Elimination Program of Kerala used the information from the Schedule H1 drug register to identify the missing TB cases and strengthen TB notification, identify providers for engagement and extend support to them for ensuring standards of TB care, and provide feedback to providers regarding prescription practices. Stakeholders felt that implementation of Schedule H1 surveillance has helped to improve TB patient notifications from the private sector, build better public-private partnerships, and improve the quality of TB diagnosis and treatment in Kerala.

**Conclusion:**

Pharmacy-based drug sales data collected either through regulatory or non-regulatory methods have immense potential to support TB elimination programs.

## Abstract (precis)

A pharmacy-based surveillance system in Kerala, India, has helped to improve TB patient notifications from the private sector, build better public-private partnerships, and improve the quality of TB diagnosis. Pharmacy-based surveillance has the potential to strengthen TB surveillance and facilitate standards of TB care.

## Teaser

**Key Findings:**

- Efforts to strengthen implementation of Schedule H1 regulation involved increasing advocacy efforts with the state government and local stakeholders, improving communication with stakeholders, educating chemists on data collection, and improving the quality of documentation of prescriptions.
- State program managers of the National TB Elimination Program used the information from Schedule H1 registers to identify missing TB cases and improve TB notification, identify providers who prescribe private anti-TB drugs and sensitize them to the Standards of TB Care in India, and sensitize providers on prescribing practices for TB.

**Key Implications:**

- Program managers may use pharmacy-based surveillance data judiciously to identify the missing TB cases, engage health care providers in prescribing practices, and improve the quality of TB diagnosis and care.
- Policy makers may realize the potential of pharmacy-based surveillance to improve TB patient notification, build public-private partnerships, and enhance standards for TB care.

## BACKGROUND

Compared to the public sector, the private health sector dominates TB care in India.^1^ A 2019 report revealed that approximately 540,000 TB cases were missing from the surveillance system across India; a major portion of missing cases were thought to be due to the gap in the notification of TB from the private sector.^2^ This raised many concerns about the quality of care that TB patients consulting the private sector in India receive.^3^^–^^4^ To improve TB care and services in the private sector, the Government of India established several policies including the Standards for TB Care in India, mandatory TB notification, and Schedule H1 drug regulation.^5^^–^^7^

To monitor the indiscriminate use of certain antibiotics and prevent the emerging threat of resistance to antimicrobial agents, in 2014, the Government of India established Schedule H1 notification, which was an amendment to the Drugs and Cosmetics Rules of 1945—the legislation that regulates the import, production, sale, prescription, and use of medicines.^6^ Schedule H1 notification controls over-the-counter sales of certain drugs, such as third and fourth generation antibiotics and psychotropic drugs, and includes 11 anti-TB drugs. The law mandates that Schedule H1 drugs can be sold by pharmacists, known as chemists in India, only on production of a valid prescription by a registered modern medicine practitioner, who has a valid qualification granted by an approved authority in the modern scientific system of medicine (excluding the homeopathic system of medicine). The chemist also needs to maintain a separate Schedule H1 register that includes the patient's identity, prescribing doctor's contact information, drug name and dispensed quantity, and date. The chemist must retain the register for at least 3 years. Each state's drugs control department is responsible for enforcing the policy.^6^

The implementation of Schedule H1 regulation has varied across the states.^8^ In most parts of the country, utilization of Schedule H1 notification was restricted to the dissemination of information. The National Strategic Plan (NSP) for TB Elimination in India 2017–2025 recognized the opportunity to strengthen the TB surveillance system by effectively implementing the Schedule H1 notification. NSP also aims at quality improvement through monitoring the quality of prescriptions. However, there were no documented systematic large-scale efforts to use Schedule H1 policy to support TB surveillance or improving the quality of care.^9^

Kerala, a state in southern India, has a population of 34.5 million. The Government of Kerala is committed to end TB and achieve Sustainable Development Goals.^10^ In 2019, Kerala had notified 68 incident TB cases per 100,000 people compared to the national notification of 159 incident TB cases per 100,000 people.^2^ In 2016, there was a felt need among the state program managers of the National TB Elimination Program (NTEP) to improve the TB surveillance in Kerala. The state program advocated with the state government to implement Schedule H1 notification more rigorously. Since 2016, the Government of Kerala has reinforced Schedule H1 implementation for anti-TB drugs as a joint venture by the drugs control department and state TB Elimination program, with monitoring from the top administrative level. In this article, we aim to document the process of implementation of the Schedule H1 surveillance to enhance the quality of TB care and strengthen the TB surveillance system in Kerala, India.

We aim to document the process of implementation of the Schedule H1 surveillance to enhance the quality of TB care and strengthen the TB surveillance system in Kerala, India.

## METHODS

We conducted 33 in-depth interviews (IDI) of people who were closely associated with the implementation of Schedule H1 including drugs control department enforcement officers (7), chemist shop owners (5), chemists' association leaders (3), NTEP district-level program managers (3), NTEP subdistrict level staff (7), private sector doctors (5), and professional medical association leaders (3). Demographic characteristics of the participants are provided in the Table. Persons to be interviewed were nominated by higher officials of the drugs control department, chemist's association leaders, leaders of professional medical associations, and the state program manager of the NTEP. Conscious efforts were taken to ensure geographical representativeness to include participants from various districts and rural and urban areas. We contacted participants by telephone, communicated the purpose of the interviews, and explained the prerequisites for the interview (stable internet connection, peaceful atmosphere, and [preferably] with video turned on). IDIs were conducted until saturation was reached and no new themes arose among each category.

**TABLE.** Demographic Characteristics of the Participants Interviewed About Schedule H1 Implementation for TB Drugs in Kerala, India

	Enforcement Officers From Drugs Control Department	Chemist Shop Owners	Leaders of Chemist's Association	NTEP Field Staff	NTEP District Program Managers	Private Sector Doctors	Professional Medical Association Leaders
Total	7	5	3	7	3	5	3
Age, years							
< 25	1	1	0	0	0	0	0
26-45	4	3	2	4	3	2	1
46-60	2	1	1	3	0	3	2
Gender							
Male	5	4	2	4	2	4	2
Female	2	1	1	3	1	1	1
Years of experience in current designation							
0–2	1	1	1	0	0	0	0
3–5	1	1	1	2	3	1	2
6–10	3	2	1	3	0	2	1
>10	2	1	0	2	0	2	0

Abbreviation: NTEP, National TB Elimination Program.

We developed an interview guide with questions to capture the evolution, process, and current status of implementation of Schedule H1 related to anti-TB drugs and the ways the NTEP used the information from the Schedule H1 register. Effective probing questions to explore specific areas of interest and additional questions to find out more about relevant issues were customized for each participant. It was piloted with 3 people: a chemist, an NTEP district-level staff person, and a private doctor. Data from pilot interviews were not used for analysis.

All interviews were conducted in the local language, Malayalam, and all except 1 interview were conducted online with the video turned on. Time was fixed based on the convenience of the participant. Other than the participant and interviewer, another researcher was present during the interview. All IDIs were conducted by A1 (male, public health expert) who was well experienced in conducting qualitative studies and was fluent in Malayalam. The presence of a female rapporteur was ensured while interviewing women. The interviewer ensured that the themes were fully discussed and that all participants were given a chance to express all their views. All interviews were conducted from January–February 2021.

The aims of the study and implications for participation were explained to all participants at the beginning of the interview. Informed consent and permission for video recording were obtained from the participants before the interviews. Confidentiality was ensured, and participants were given a chance to opt out freely at that stage without giving any reason. All but 1 private-sector doctor contacted participated. Two participants called and discussed additional points after formal interviews. Each interview lasted for approximately 40 minutes (range 23 minutes–65 minutes).

IDIs were later transcribed verbatim and translated into English. One researcher recorded the proceedings, identifying key themes and monitoring verbal and nonverbal interactions by watching the video recordings. The transcripts were then manually coded by 2 researchers and emerging themes and subthemes were identified. Sections with similar coding were grouped according to the predetermined themes. Repeated themes were marked as important in red. All the flagged statements were put together and synthesized. The team read the transcripts and notes and reached a consensus. Any disagreements were discussed regularly within the team to reach a consensus regarding theme coding. Important responses were quoted, which evoked spontaneous discussion, around which a lot of time was spent and had some emotional cues attached. Annual NTEP program performance reports were checked to obtain quantitative information related to notification from the private sector and quality of care indicators. Quantitative information from the report published by the NTEP at Kerala related to anti-TB drug sales were also captured. Information obtained through qualitative interviews was corroborated with the quantitative information available.

Ethics approval was obtained from the Independent Ethics Committee of Centre for Public Health Protection (IEC-CPHP-2019-10/12), Kerala, India.

## RESULTS

We have compiled the findings of the IDIs into 2 categories: implementation of Schedule H1 and ways the NTEP used the Schedule H1 information.

### Implementation Process of Schedule H1 Surveillance

#### 1. NTEP Advocacy With State Government

To strengthen TB surveillance and to ensure that public health authorities are notified about all patients, NTEP TB program managers convinced the state administrators to effectively implement Schedule H1 notification related to anti-TB drugs with monitoring mechanisms from the highest administrative level. State government orders were issued for joint visits to chemist shops by NTEP key staff and drugs control department enforcement officers in November 2016, making both the departments accountable. The purpose of their visit was to educate the chemist shops regarding the Schedule H1 register and facilitate mandatory TB notification with their support. Quarterly reviews were conducted by the state health authority with the drugs control department and NTEP state program manager.

#### 2. Information, Education, and Communication of Chemists

NTEP and the drug control department jointly convened bi-annual meetings at the district level of chemist's associations, chemist's shop owners, and private chemists to highlight the importance of maintaining the Schedule H1 register for anti-TB drugs and conducted annual sensitization sessions for chemist shop owners and chemists. Posters regarding the need to maintain the information of patients were prepared and publicly displayed in all chemist's shops. WhatsApp groups were formed regionally by the district-level staff of NTEP including mapped chemists, drug enforcement officials, and chemist association leaders specifically for better communication related to the implementation of Schedule H1 and addressing queries of chemists related to NTEP services.

Posters regarding the need to maintain the information of patients were prepared and publicly displayed in all chemist's shops.

#### 3. Rapport and Exchange of Information

The enforcement officers regularly collected the list of chemist's shops that stocked/sold anti-TB drugs from the distributors, mapped the shops, and kept the list updated. They shared the information with NTEP district program managers. Of the approximately 15,000 private chemist outlets in Kerala, only 650 stocked or sold anti-TB drugs according to the latest list. NTEP staff at the subdistrict level were asked to visit these chemist shops monthly and maintain a good rapport with them. Maintaining good rapport between NTEP staff and the chemists made the process of information exchange easier. Contact information was exchanged mutually. The chemist shops provided digital and print copies of the monthly consolidated Schedule H1 reports to NTEP staff.

#### 4. Review of Schedule H1 Surveillance Activities

During monthly meetings at the district level, NTEP reviewed the staff efforts to collect Schedule H1 information. Each staff explained the process indicators, such as the number of chemist shops stocking anti-TB drugs, number of shops visited that month, details of cases not found in NIKSHAY (the case-based online management information system of NTEP)^11^ but found in the Schedule H1 register, and follow-up actions required. Drugs control department enforcement officers were also invited by the NTEP district program managers while reviewing Schedule H1 surveillance activities at the monthly review meetings. Visiting chemist shops was also part of periodic internal evaluations of NTEP conducted from the state level.

*NTEP district program manager used to check our tour diary and see how many chemist shops we have visited. We need to present the information that we obtained from Schedule H1 and its status in every monthly NTEP review meeting.* —NTEP district-level staff

#### 5. Advocacy Campaigns

Every year, 1-week long state-wide campaigns have been conducted to reinforce the importance of maintaining Schedule H1 registers. A dedicated team comprising 62 drugs control department enforcement officers, 127 NTEP key staff, and 42 chemist's association representatives visited the mapped chemists' shops to reeducate them on the importance of maintaining the Schedule H1 register, verify the status of maintenance of Schedule H1 register, ensure that details of all anti-TB drug sales were conveyed to the local program managers of NTEP, provide onsite feedback and support to them in case of any gaps identified. The team also displayed public education materials related to Schedule H1 at chemists' shops.

#### 6. Quality Improvements in Documentation

Because of limited staff and high customer turnover, it was difficult to document all patient information at the chemist shops. Chemist's association leaders opined that more than 90% of chemist shops were using computer-generated bills. Most of the chemist shops used the billing software developed by 7 private companies. They also reported that based on the felt need by chemists, the billing software of approximately 70% of chemist shops had been modified by the software developers to select Schedule H1, if applicable, against each sale. Provisions for autogenerated Schedule H1 reports were made in the billing software itself, a solution introduced by the billing software developers.

Some chemists described unclear prescriptions by doctors as a challenge for completing the information in the Schedule H1 register. Chemists reported that the doctor's name was missing in approximately 10%–20% of prescriptions.

*Prescriptions will not clearly mention the names of doctors. They will only have designations especially those coming from medical colleges. Patient will also find it difficult to track the doctor if we send him/her back. We don't want patient to suffer. So, we issue the drugs leaving that [name of doctor] column blank.* —Chemist's association leader

Some chemists described unclear prescriptions by doctors as a challenge for completing the information in the Schedule H1 register.

NTEP program managers discussed the issue of unclear prescriptions with hospitals and professional medical associations, which then facilitated communication to all their members to write clear prescriptions with the doctor's name.

Frequent meetings with the chemists and the chemist's association by district authorities helped in devising local solutions and resulted in improving the quality of the documentation.

#### 7. Enforcements

Most of the efforts were focused on education, communication, and quality improvements. Drug enforcement officials warned many chemists of imposing penalties in case of noncompliance; however, no one imposed any penalties in this regard to date.

### Ways NTEP Used the Information From Schedule H1

Initially, in 2016, NTEP at Kerala tried to enter the information received from the Schedule H1 register directly to NIKSHAY. However, staff identified 3 challenges to this process of feeding the information directly to NIKSHAY.

1. Chemists found it difficult to collect patients' contact information accurately. NTEP program managers at the district level reported that approximately 20%–25% of the patients identified through the Schedule H1 register could not be contacted because of incomplete/incorrect contact information.
2. NTEP program managers at the district level also reported that approximately 5%–10% of patients who were prescribed anti-TB drugs did not have TB. They were prescribed the drugs for other conditions, such as staphylococcus bone infections, urinary tract infections, or as chemoprophylaxis against non-tuberculous conditions.
  *When we call patient, they become really upset. They may not have TB. They might have been prescribed this for something else. It has led even to open complaints against us by the patient*. —NTEP district-level staff
3. Patients purchased medicines from different chemist shops and different districts during anti-TB treatment, making it difficult to eliminate duplication of information.
  *Out of enthusiasm, I notified all the cases that I obtained through Schedule H1 from chemist shop in 2016. Then only I understood that some of them were not really having TB, some of them were already notified in the neighboring districts, many of them could not be contacted due to incomplete information. That year the entire districts treatment success rate went down*. —District TB program manager

After these initial experiences, the practice of notifying TB directly from the Schedule H1 register was stopped. Based on the initial experiences, from 2018 onward, the state program managers of NTEP evolved their own Schedule H1 surveillance system. NTEP at Kerala used the information from Schedule H1 for 3 purposes (Figure).

**FIGURE:**
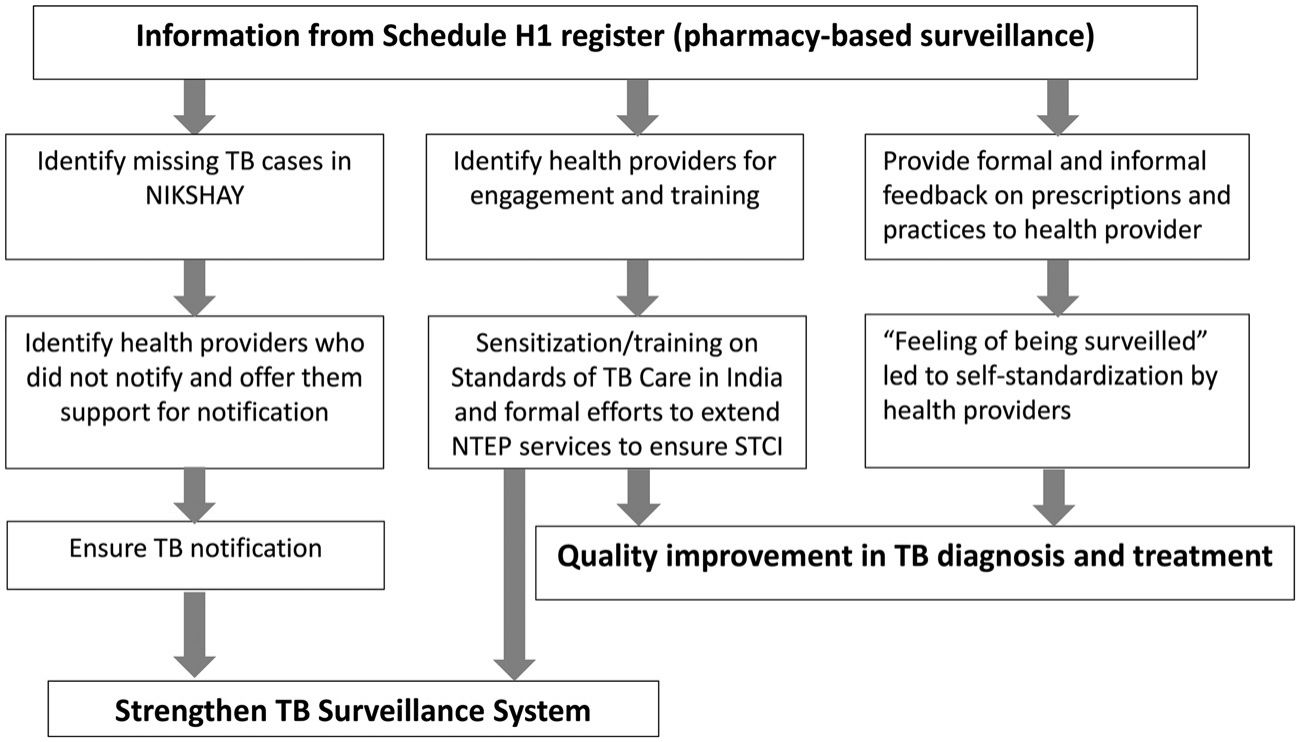
How Did the National TB Elimination Program Use Pharmacy-Based Surveillance Data?

#### 1. Identify the Missing TB Cases and Strengthen TB Notification

To identify missing cases, NTEP staff verified NIKSHAY data against the consolidated Schedule H1 register and wrote a NIKSHAY identification number next to each name in the register. They contacted the health providers of cases that did not have an identification number to get the additional information and offered support to the providers to complete the notification.

*In 2018, 18 TB cases that we identified form Schedule H1 were not in NIKSHAY. It was 3 doctors who treated those 18 cases. We met those doctors, sensitized them about mandatory TB notification, and offered them support for notification. Last year, we got only 2 cases from Schedule H1 surveillance that were not in NIKSHAY.* —District TB program manager

*Based on Schedule H1 data, I used to write friendly letters to doctors who did not notify TB offering them support for notifications. Now they inform all TB cases the moment they diagnose*. —District TB program manager

*Private sector notifications improved, almost doubled over last 2 years, directly from private doctors and hospitals in my district after we implemented Schedule H1. Doctors and hospitals now knew that we have a mechanism to identify if they have not notified. They now understood that we are serious about it.* —District TB program manager

#### 2. Identify Private-Sector Providers for Engagement and Extending Support for Ensuring Standards of TB Care in India (STCI)

NTEP identified the providers who prescribed private anti-TB drugs and then sensitized/trained them in STCI. NTEP offered the providers free drugs including TB preventive therapy for eligible contacts, free diagnostics, support for contact investigations, and linkages to social welfare schemes to help providers ensure STCI to their patients.

*Schedule H1 surveillance has definitely helped us in engaging private sector. We could identify all providers who deals with TB and directly talk to them. 90% of those doctors are now prescribing NTEP drugs.* —District TB program manager

*From Schedule H1 surveillance we identified that a good number of prescriptions are being sent to private chemist shops from government medical colleges especially from surgical and super specialty departments. That was mainly because of their ignorance about the NTEP. Through medical college core committee, we organized formal letters and sensitization sessions targeting them. The prescriptions from government medical colleges have come down now*. —District TB program manager

*Most of the doctors were more willing to offer NTEP drugs now to the patients. Only in some circumstances like patients who want to travel abroad or patients who insists on private drugs are being prescribed private anti-TB drugs. Implementation of Schedule H1 has played a major role in this attitude change.* —Private sector doctor

*Sale of anti-TB drugs dropped like anything over last 3 years. Very few doctors are now prescribing private anti-TB drugs. Very few chemists are stocking and selling it*. —Drug control department enforcement officer

#### 3. Provide Feedback to Doctors Regarding Prescription Practices

District TB program managers provided feedback to doctors about prescribing practices directly or indirectly through professional medical associations.

*We have projected a few prescriptions after removing all identities during doctor's meeting.* —Professional association leader

*We have observed a sudden cluster of cases in an area through Schedule H1 register. When we investigated all those were prescribed anti-TB drugs by a single pediatrician who recently settled in our area. We met him, sensitized about diagnostic algorithm, made him attended a training on pediatric TB. We made arrangements for free GeneXpert test for his patients. Now he uses anti-TB drugs judiciously*. —District TB program manager

### Quality of TB Diagnosis and Treatment Improved as a Byproduct

NTEP program managers, professional association leaders, and private-sector doctors agreed that there was a decrease in the use of empirical anti-TB drugs by clinicians for patients who did not have a definitive diagnosis and there was an increased effort for obtaining a definitive diagnosis of TB before initiation of anti-TB drugs. Private-sector doctors mentioned a “self-standardization” in the practice of diagnosing and treating TB and an effort to follow standards of TB care.

*I feel the major drop in sales was due to decline in the practice of doctors prescribing empirical [anti-TB drug]. Now they think twice before prescribing an empirical [anti-TB drug]*. —Private-sector doctor

*We have noticed an improvement in quality of TB prescriptions. Quinolones were being prescribed with many of the TB prescriptions previously. Now we could not find many.* —Chemist

### Other Insights During the Implementation of Schedule H1 Surveillance

Apart from the above-mentioned uses, the NTEP program received other insights regarding the use of anti-TB drugs, including the use of anti-TB drugs to treat TB in elephants. This led to further investigations into the magnitude of zoonotic TB.

*I have noticed unusually high proportion of anti-TB drug sales from a chemist shop which is not matching with records. On further investigation, I found that the drugs were being used for treatment of TB among elephants. That was a new insight*. —Drug control department enforcement officer

No differences were noticed among the experiences of providers from urban or rural districts.

### Quantitative Data From the Program Reports

The state drugs control department regularly collects the details of the sale of rifampicin-containing products from all drug companies selling anti-TB drugs in the state. State program managers of NTEP have analyzed the drug sales data and reported that anti-TB drugs sales in Kerala have decreased by 70% in 2019 compared to 2015.^12^ Official TB surveillance data of the state showed an increase in TB notifications from the private sector in Kerala (2018: 3981; 2019: 4,927; 2020: 5,795).^2^ The proportion of microbiologically confirmed cases among TB notified cases from the private sector increased from 25% in 2018 to 34% in 2019 to 38% in 2020.^2^ The estimated number of unnotified TB cases per 100,000 people based on the total sales of rifampicin-containing products in Kerala showed an annual decline of 22% in last 3 years, closing the gap in the surveillance system.^13^

## DISCUSSION

The article describes the process of implementing the Schedule H1 system to enhance the quality of TB care and strengthen the TB surveillance system in Kerala, India. The information obtained from the qualitative interviews corroborated with the official data in terms of increase in notification from the private sector, decline in private anti-TB drug sales, and increased efforts for obtaining a microbiological confirmation for TB in the private sector. For the process of subnational certification for TB elimination by the Government of India, an independent verification agency surveyed 83,000 individuals who were selected using a multistage random sampling in Kerala in February 2021 to identify missing TB cases in the community.^14^ The survey revealed that there were no missing TB cases in the community. Schedule H1 surveillance may not be the only reason for this finding, but the surveillance has helped the state to identify TB cases missing from the surveillance system and close the gaps. It also helped the NTEP to identify the correct providers for engaging. Although it is a regulatory tool, Schedule H1 was used to build partnerships between NTEP and the private health care providers.

Schedule H1 surveillance may not be the only reason that missing cases have been identified, but the surveillance system has helped identify TB cases and close the gaps.

A study done in another state in southern India showed that pharmacy-based surveillance has identified approximately one-fourth of the total TB patients notified.^15^ There are many efforts from other Indian states of Chhattisgarh and Punjab to use Schedule H1 to support TB surveillance. Schedule H1 may provide a good opportunity to support TB surveillance if effectively executed and information of prescription details is used.

The national strategy for TB elimination in India has envisioned prescription audits based on Schedule H1 information for quality improvement.^16^ Experiences from a private tertiary care center in Kerala showed that establishing a peer audit system resulted in decreasing the number of inappropriate prescriptions from 38% (2017–2018) to 18% (2018–2019).^16^ Prior implementation of a lung health project in a primary health care setting in Kerala also demonstrated that the presence of standards, good quality trainings, and monitoring of prescriptions have trimmed the antibiotic use by 50% among patients with chronic respiratory diseases over a year.^17^ These experiences show that incorporating the prescription audit for improving the quality of TB diagnosis and treatment is meaningful. Kerala plans to establish a systematic peer audit system with the help of a coalition of professional medical associations to further improve the quality of TB diagnosis and treatment.

Although the Government of India established the Schedule H1 act in 2014, the Government of Kerala only started enforcing it systematically for anti-TB drugs after advocacy by the NTEP. Enforcement for other drugs in Schedule H1 is still suboptimal in the state. In Kerala, most of the chemist shops used computer-generated bills so incorporation of digital solutions was possible. However, those solutions may not be possible in rural parts of other states that have manually maintained records. The number of chemist shops selling anti-TB drugs was also limited in Kerala due to a lower disease burden and good public-private partnerships, so coordination and managing information was easy. In high-burden settings, an information system, such as a uniform electronic tool to capture the information that interacts with the NTEP database, could be considered. In such scenarios, capacity needs to be built among program managers to establish systems for decentralized compilation and use of information locally for appropriate actions. It should be noted that surveillance of prescriptions and Schedule H1 register and backtracking based, if not done in a mutually agreeable and professional manner, may threaten or erode the trust of the private sector “fraternity.”

Experiences from the current study may be helpful for settings working to strengthen their TB surveillance system to reach Sustainable Development Goals/End TB targets and United Nations high-level meeting targets or trying to engage private sectors/trying to improve standards of TB care. Pharmacy-based drug sales data collected either through regulatory or nonregulatory methods have immense potential to support TB elimination programs.

## Notes

**Peer Reviewed**

**First published online:** October 28, 2021.
